# Antitumor effects of the investigational selective MEK inhibitor TAK733 against cutaneous and uveal melanoma cell lines

**DOI:** 10.1186/1476-4598-11-22

**Published:** 2012-04-19

**Authors:** Erika von Euw, Mohammad Atefi, Narsis Attar, Connie Chu, Sybil Zachariah, Barry L Burgess, Stephen Mok, Charles Ng, Deborah JL Wong, Bartosz Chmielowski, David I Lichter, Richard C Koya, Tara A McCannel, Elena Izmailova, Antoni Ribas

**Affiliations:** 1 Department of Medicine, Division of Hematology/Oncology, University of California Los Angeles (UCLA), Los Angeles, CA, USA; 2 Department of Ophthalmology, The Jules Stein Eye Institute, UCLA, Los Angeles, CA, USA; 3 Jonsson Comprehensive Cancer Center at UCLA, Los Angeles, CA, USA; 4 Jonsson Comprehensive Cancer Center at UCLA, Los Angeles, CA, USA; 5 Millennium Pharmaceuticals, Inc., Cambridge, MA, USA; 6 Division of Hematology-Oncology, 11–934 Factor Building, UCLA Medical Center, 10833 Le Conte Avenue, Los Angeles, CA, 90095-1782, USA

**Keywords:** Melanoma, MEK inhibitor, BRAF mutation, Oncogenes, MAPK signaling

## Abstract

**Background:**

TAK733 is a novel allosteric, non-ATP-binding, inhibitor of the BRAF substrates MEK-1/2.

**Methods:**

The growth inhibitory effects of TAK733 were assessed in a panel of 27 cutaneous and five uveal melanoma cell lines genotyped for driver oncogenic mutations. Flow cytometry, Western blots and metabolic tracer uptake assays were used to characterize the changes induced by exposure to TAK733.

**Results:**

Fourteen cutaneous melanoma cell lines with different driver mutations were sensitive to the antiproliferative effects of TAK733, with a higher proportion of *BRAF*^*V600E*^ mutant cell lines being highly sensitive with IC50s below 1 nM. The five uveal melanoma cell lines had GNAQ or GNA11 mutations and were either moderately or highly sensitive to TAK733. The tested cell lines wild type for *NRAS, BRAF, GNAQ* and *GNA11* driver mutations were moderately to highly resistant to TAK733. TAK733 led to a decrease in pERK and G1 arrest in most of these melanoma cell lines regardless of their origin, driver oncogenic mutations and *in vitro* sensitivity to TAK733. MEK inhibition resulted in increase in pMEK more prominently in *NRAS*^*Q61L*^ mutant and *GNAQ* mutant cell lines than in *BRAF*^*V600E*^ mutant cell lines. Uptake of the metabolic tracers FDG and FLT was inhibited by TAK733 in a manner that closely paralleled the *in vitro* sensitivity assays.

**Conclusions:**

The MEK inhibitor TAK733 has antitumor properties in melanoma cell lines with different oncogenic mutations and these effects could be detectable by differential metabolic tracer uptake.

## Introduction

Most melanomas have mutually-exclusive activating mutations in the mitogen-activated protein kinase (MAPK) pathway involving *NRAS* or *BRAF* genes in melanomas of skin primary, *c-Kit* in acral and mucosal melanomas, and *GNAQ* and *GNA11* in uveal melanomas [1-5]. These mutations render melanoma cells independent of the normal receptor tyrosine kinase (RTK)-mediated pathway regulation, and constitutively drive melanoma cells to oncogenic proliferation and survival [6]. The most common of these mutations is the *BRAF*^*V600E*^ mutation, present in approximately 50% of melanomas of skin origin. *BRAF*^*V600E*^ mutant cutaneous melanomas are dependent on MAPK signaling for cell-cycle progression and proliferation, and have high sensitivity to type I BRAF inhibitors and to MEK inhibitors [7-10]. Very high response rates and improved survival have been noted with the administration of the type I BRAF inhibitor vemurafenib (formerly PLX4032/RG7204) to patients with BRAF^V600E^ mutant cutaneous metastatic melanoma [11-13]. Tumor responses were dependent on the presence of the *BRAF*^*V600E*^ oncogene and efficient inhibition of the MAPK pathway as detected by decreased phosphorylation of ERK [8]. Inhibition of the immediately downstream MEK1/2 kinases in BRAF^V600E^ mutant cutaneous melanoma was shown to lead to marked inhibition of cell proliferation in cell lines [7]. The attractiveness of inhibiting at the level of MEK is supported by the very high kinase specificity of allosteric MEK inhibitors and the fact that MEK1/2 kinases are critically positioned as a funnel in the MAPK pathway downstream of the three RAS isoforms and the three RAF isoforms. Therefore, the inhibition of MEK1/2 with specific MEK inhibitors may result in blocking MAPK signaling from multiple upstream oncogenes. Preclinical studies suggest that some *NRAS*-mutant cutaneous melanomas may also exhibit sensitivity to RAF or MEK inhibition [14], whereas *KRAS* mutations have conferred only marginal sensitivity [15]. Gene expression profiling studies mapping the gene signatures downstream of a constitutively activated MAPK pathway suggested that cutaneous melanoma cell lines with NRAS mutations are less dependent in signaling through this pathway compared to *BRAF*^*V600E*^ mutant cutaneous melanoma cell lines [10,16], explaining in part the differential sensitivity of NRAS and BRAF mutant cells to MEK inhibitors [7].

*BRAF* and *NRAS* mutations are absent in melanomas arising in the uveal layer of the eye, but mutually exclusive somatic mutations in the heterotrimeric G protein alpha-subunit, GNAQ, or in GNA11, are present in the great majority of uveal melanomas [4,5]. It had long been noted that uveal melanomas have constitutive MAPK signaling [17,18], and it is now understood that it is because of the presence of GNAQ or GNA11 mutations. These mutations occur in codons 183 or 209 in the Ras-like domain and result in constitutive activation, turning the GNA proteins into dominant-acting oncogenes signaling through the MAPK pathway [4]. GNAQ knockdown, as well as treatment with the U0126 MEK inhibitor, resulted in inhibition of MAPK signaling and loss of viability [4]. Therefore, MEK inhibition may be a way to treat metastatic melanoma of uveal origin, a disease that has been highly refractory to most therapies tested to date.

TAK733 represents a novel and distinct inhibitor of MEK that is capable of allosteric inhibition of the RAF substrates MEK-1 and MEK-2 [19]. This compound has been characterized extensively and shown to possess desirable drug-like attributes [20]. In the current studies we have analyzed the sensitivity and resistance of human cutaneous and uveal melanoma cell lines to this novel MEK inhibitor, with analysis of the oncogenic driver mutations and downstream signaling alterations and functional effects.

## Results

### Sensitivity of cutaneous and uveal melanoma cell lines to TAK733

**Figure 1:**
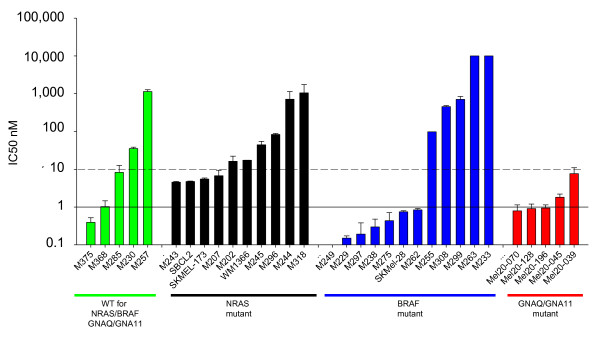
**TAK733 half maximal inhibition concentration (IC50) values as a function of*BRAF, NRAS, GNAQ*or*GNA11*mutational status.** The cells were treated for 72 hours and cell viability was determined by MTS colorimetric assay. IC50 values (x-axis) are expressed in nM for TAK733. Error bars represent standard error of the mean.

Cutaneous and uveal melanoma cell lines were cultured *in vitro* in the presence of increasing concentrations of TAK-733 for 72 hours to determine the half maximal inhibitory concentration (IC50) in cell proliferation assays. Cell lines with an IC50 less than 10 nM were considered sensitive, and cell lines with IC50 less than 1 nM were considered highly sensitive. Among 12 *BRAF*^*V600E*^ mutated cutaneous cell lines tested, seven were highly sensitive to TAK-733 with IC50s less than 1 nM (Figure 1 and Additional file 1: Figure S1). Five *BRAF*^*V600E*^ mutant cutaneous cell lines had an IC50 higher than 100 nM and were considered highly resistant to this agent.. Among ten *NRAS*^*Q61*^ mutant cutaneous melanoma cell lines, four were sensitive with IC50s below 10 nM, but none was highly sensitive. Our panel also included five cutaneous melanoma cell lines wild type for mutations in *NRAS, BRAF, GNAQ* and *GNA11* and only one was highly sensitive to TAK733 with IC50s below 1 nM, while two were considered sensitive with IC50 less than 10 nM. All five uveal melanoma cell lines were sensitive to TAK733 with IC50 values below 10 nM, with three of them being highly sensitive. All these cell lines carried *GNAQ* or *GNA11* driver mutations (Figure 1 and Table 1). Overall, there was a clear trend of higher sensitivity in *BRAF* mutant cell lines, but all subgroups included cell lines with variable sensitivity and also high resistance to exposure to the MEK inhibitor.

**Table 1:** Characterization of oncogenic alterations in melanoma cell lines tested for sensitivity to TAK733

**Driver Oncogenic Mutation**	**Cell Line**	**Known Oncogenic Alterations**
Wild type for BRAF and NRAS	M230	*KIT L576P*
	M257	*CDKN2A* R80
		*BRAF* amplification
	M285	None detected
	M368	None detected
	M375	None detected
NRAS Mutants	M202	*NRAS*^*Q61L*^
		*EGFR* amplification
		*CDKN2A* homozygous deletion
	M207	*NRAS*^*Q61L*^
		*MITF* amplification
		*EGFR* L747_P753 > S
		*PTEN* heterozygous deletion
	M243	*NRAS*^*Q61H*^ homozygous
		*PTEN*^E156G^ heterozygous
		CTNNB1_D32Y
	M245	*NRAS*^*Q61K*^ heterozygous
		*TP53*^*R273H*^
	M244	*NRAS*^*Q61K*^ heterozygous
	M296	*NRAS*^*Q61R*^ heterozygous
	M318	*NRAS*^*Q61L*^ heterozygous
		*PIK3CA*^*C420R*^
	SBCL2	*NRAS*^Q61K^ homozygous
	Wn1366	*NRAS*^Q61L^ heterozygous
BRAF mutants	M229	*BRAF*^V600E^ homozygous
		*BRAF* amplification
		*MITF* amplification
		*AKT1* amplification
		*PTEN* heterozygous deletion
	M233	*BRAF*^*V600E*^ heterozygous
		*BRAF* amplification
		*AKT1* amplification
		*CCND1* amplification
		*EGFR* amplification
		*CDKN2A* homozygous deletion
		*PTEN* homozygous deletion
	M238	*BRAF*^*V600E*^ heterozygous^1^
		*CDKN2A* homozygous deletion
		*PTEN* heterozygous deletion
	M249	*BRAF*^*V600E*^ heterozygous
		*BRAF* amplification
		*MITF* amplification
		*AKT2* amplification
		*PTEN* homozygous deletion
	M255	*BRAF*^*V600E*^ heterozygous
		*AKT2* amplification
		*CCND1* amplification
		*EGFR* amplification
		*CDKN2A* homozygous deletion
	M262	*BRAF*^*V600E*^ homozygous
		*AKT1* E17K
		*AKT1* amplification
		*EGFR* amplification
		*CDKN2A* homozygous deletion
	M263	*BRAF*^*V600E*^ heterozygous
		*CDKN2A* heterozygous deletion
	M275	*BRAF*^*V600E*^ heterozygous
	M297	*BRAF*^*V600E*^ homozygous
		*PTEN*^*G165X*^ homozygous
	M299	*BRAF*^*V600E*^ heterozygous
	M308	*BRAF*^*V600E*^ heterozygous
		*BRAF* amplification
		*MITF* amplification
		*AKT2* amplification
		*EGFR* amplification
		*CDKN2A* heterozygous deletion
	SKmel28	*BRAF*^*V600E*^ homozygous
		*EGFR*^*P753S*^
		*MITF* amplification
		*CCND1* amplification
		*CDKN2A* heterozygous deletion
		*PTEN* heterozygous deletion
GNAQ or GNA11	Mel20-06-039	*GNAQ*^*Q209L*^ heterozygous
	Mel20-06-045	*GNAQ*^*Q209P*^ heterozygous
	Mel20-07-070	*GNA11*^*Q209L*^ heterozygous
	Mel20-08-128	*GNA11*^*Q209L*^ heterozygous
	Mel20-09-196	*GNAQ*^*Q209P*^ heterozygous

Legend: ^1^Tandem mutation where the codon changed from GTG to GAA.

### TAK733 has similar inhibitory effects on cell cycle in sensitive and resistant cutaneous melanoma cell lines

**Figure 2:**
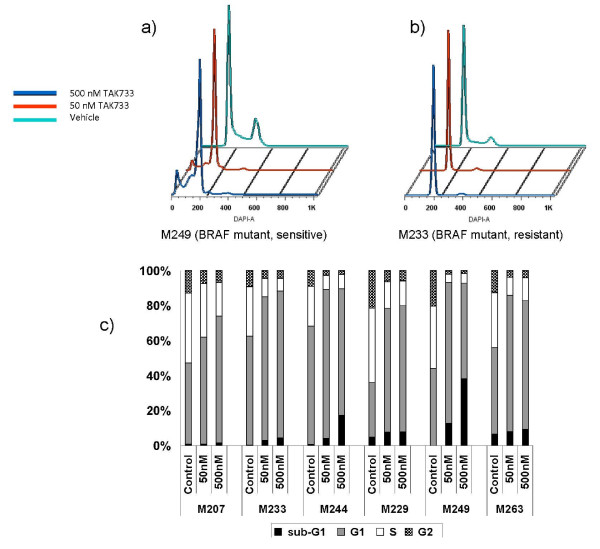
**Effects of TAK733 on cell cycle.a** and **b**) Examples of the flow histograms of a sensitive and a resistant cutaneous melanoma cell line. **c**) Bar graph of G1, S and G2 phase as percent change from baseline. Six melanoma cell lines representative of the spectrum of sensitivities of *NRAS* mutants (sensitive: M207; resistant: M244) and *BRAF* mutants (sensitive: M229 and M249; resistant: M233 and M263) were cultured with 50 nM and 500 nM of TAK733 for 48 hours and stained with DAPI for cell cycle analysis.

To study the effects of TAK733 on cell cycle progression downstream of MEK signaling we used DAPI flow cytometric staining (representative examples of flow histograms in Figure 2a and b). For these studies we chose two *NRAS* mutants and four *BRAF* mutants that represented the spectrum of sensitivities of these cell lines. The *NRAS* mutants M207 (sensitive) and M244 (highly resistant) both had a dose-dependent G1 arrest with increasing concentrations of TAK733 (Figure 2c). The same was evident with the four *BRAF* mutants, including the two with high sensitivity (M229 and M249) and the highly resistant (M233 and M263). The sub-G1 peak also did not predict the cell proliferation assay results, even though the sharpest increase was in M249, one of the most sensitive cell lines (Figure 2 a and c). Overall, TAK733 exposure for up to 48 hours led to a similar G1 arrest in melanoma cell lines regardless of their origin, driver oncogenic mutations and *in vitro* sensitivity to TAK733).

### Modulation of MAPK and PI3k/akt signaling pathways upon exposure to TAK733

**Figure 3:**
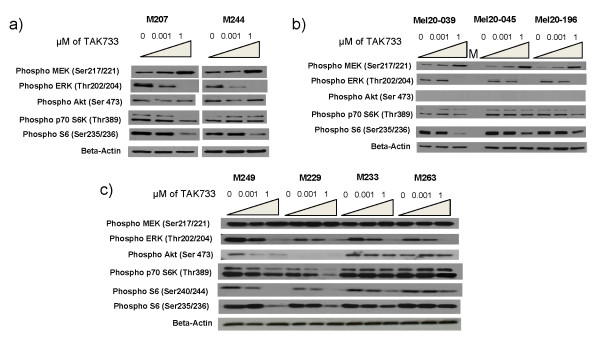
**Effects of TAK733 on the signaling of the MAPK and PI3K/AKT pathways by Western blot analysis.** Melanoma cell were exposed for 24 hours to solvent (DMSO) or various concentrations of TAK733. **a**) *NRAS*^*Q61*^ mutated cutaneous melanoma cell lines; **b**) *GNAQ* or *GNA11* mutated uveal melanoma cell lines; **c**) *BRAF*^*V600E*^ mutated cutaneous melanoma cell lines.

To explore how cell lines with different mutations respond differently to TAK733 we analyzed signaling pathways in representative cell lines with similar growth kinetics but with markedly different sensitivities to TAK733. Among the *NRAS*^*Q61L*^ mutant cutaneous group we chose the resistant M244 and the sensitive M207. Among the *BRAF*^*V600E*^ mutant cutaneous group we chose M229 and M249 as representatives of highly sensitive cutaneous cell lines, and M233 and M263 as resistant cutaneous cell lines. In our panel, all the uveal melanoma cell lines were sensitive to TAK733 and we picked three as representative samples with *GNAQ* mutations. As expected based on prior data [21], MEK inhibition resulted in increase of pMEK in non-*BRAF*^*V600E*^ mutant cell lines (Figure 3). This was more prominent in *NRAS*^*Q61L*^ mutant and uveal melanoma cell lines than in *BRAF*^*V600E*^ mutant cell lines, which had a higher baseline level of pMEK. In all cases, TAK733 induced a marked dose-dependent decrease of pERK, regardless of the driver oncogenic mutation or the sensitivity or resistance to this agent in cell viability assays. On the contrary, effects on pAKT and pS6K varied according to the cell origin, oncogenic events and sensitivity to TAK733. *BRAF*^*V600E*^ mutant cell lines resistant to TAK733 showed no inhibition of pAKT or pS6K, while there was a general trend towards inhibition of these two phosphorylated molecules in sensitive cell lines. Of note, in the uveal melanoma cell lines and in the cutaneous melanoma cell line M229, the baseline level of pAKT was undetectable by Western blot, so no inhibition could be recorded in them. Changes in pS6 tended to follow changes in pS6K in the cutaneous melanoma cell lines but not in the uveal melanoma cell lines. In a time-course analysis of signaling events upon exposure to TAK733, both the sensitive M229 and the resistant M233 cell lines with *BRAF*^*V600E*^ mutations showed initial inhibition of pERK, but the resistant cell line recovered pERK signaling with time (Additional file 2: Figure S2). This different time-course effect was not evident for the inhibition of pAKT or pS6K in the resistant cell line, while they were permanently inhibited over the 48 hour study period in the sensitive cell line.

### Differential metabolic tracer uptake between cell lines sensitive and resistant to TAK733

**Figure 4:**
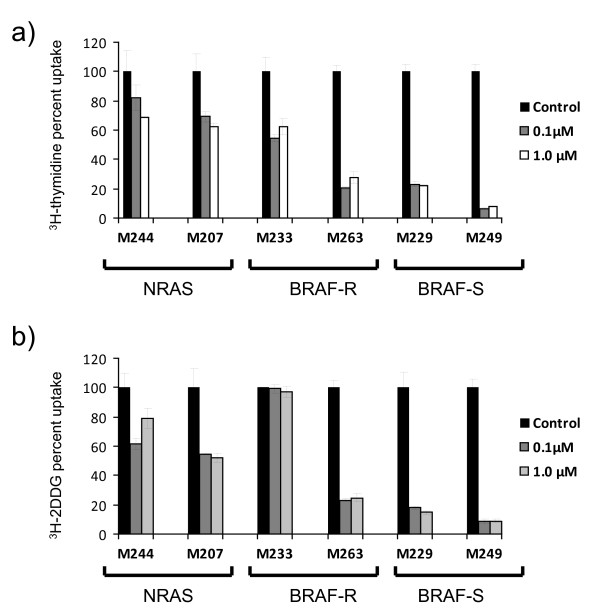
**Metabolic tracer uptake profile upon exposure to TAK733.** The same six melanoma cell lines from Figure 2 representing the spectrum of sensitivities for *NRAS* and *BRAF* mutant cells were exposed to TAK733 and the relative metabolic tracer uptake was calculated compared to cultures exposed to DMSO vehicle control. **a**), [^3^H]-2DDG. **b**) [^3^H]-thymidine.

We explored the use of metabolic tracers to differentiate response or resistance to TAK733 in six cutaneous melanoma cell lines with the goal of a future use of these tracers in PET scanning studies in the clinic. Thymidine is taken up by proliferating cells and the PET tracer [^18^ F]FLT can be used in patients. Consistent with the cell cycle analysis data, all the tested cell lines had some degree of inhibition of tritium-labeled thymidine (^3^H-thymidine) uptake upon exposure to TAK733 regardless of their sensitivity *in vitro*. The highest levels of inhibition were in the highly sensitive *BRAF*^*V600E*^ mutant cell lines M229 and M249 and the relatively resistant M263 cell line (Figure 4a). Changes in uptake of tritium-labeled 2'-deoxy-D-glucose (^3^H-2DDG) were analyzed to study effects of TAK733 on PET scans with the commonly used PET tracer [^18^F]FDG. The lowest degree of inhibition was in the two most resistant cell lines, the *BRAF*^*V600E*^ mutant M233 and the *NRAS*^*Q61K*^ mutant M244 (Figure 4b). Therefore, changes in the uptake of the ^3^H-2DDG metabolic tracer most closely followed the results of the cell viability assays.

## Discussion

Initial data testing MEK inhibitors in melanoma cell lines suggested a high level and selective sensitivity in *BRAF*^*V600E*^ mutant melanoma cell lines, with low sensitivity in melanoma cell lines with other driver oncogenes [7]. Further testing with expanded panels of cell lines has confirmed a trend towards higher sensitivity in *BRAF*^*V600E*^ mutant melanoma, but has also provided evidence that some melanoma cell lines with *NRAS* activating mutations are sensitive to MEK inhibitors [10,14]. The higher sensitivity of *BRAF* mutant cell lines compared to *NRAS* mutant cell lines is generally represented in our series, but some *BRAF* mutants have high resistance to the MEK inhibitor while some *NRAS* mutants are sensitive. It is certainly possible that our *BRAF*^*V600E*^ mutant cutaneous melanoma panel is skewed for cell lines with natural resistance to inhibition of the MAPK pathway, since we have previously reported a similar greater than expected frequency of cutaneous cell lines resistant to the type I BRAF inhibitor vemurafenib [9,22]. The molecular basis for this relative high frequency of natural resistance of *BRAF*^*V600E*^ mutant cutaneous melanoma cell lines in our series is currently not well understood. Initial exploration of secondary oncogenic events in the PI3K/AKT pathway (such as PTEN deletions) did not clearly differentiate naturally sensitive and resistant *BRAF*^*V600E*^ mutant cutaneous melanomas to the BRAF inhibitor vemurafenib, but downstream signaling studies did suggest that the PI3K/AKT pathway may be involved [9,22]. In the current studies we noted the same phenomenon, a lack of correlation between natural sensitivity and resistance to TAK733 based solely on oncogenic analysis of the cell lines using SNP arrays or targeted oncogene sequencing for mutations frequently present in cancer. However, there was a suggestion from Western blot analyses of signaling pathways that differential effects of MEK inhibitor altering signaling through the PI3K/AKT pathway may be related to resistance. This observation may provide means to explore combinations of MEK inhibitors with PI3K or AKT inhibitors that may be useful in *NRAS* or *BRAF* mutant melanomas, which could be due to hyperactive receptor tyrosine kinase signaling leading to resistance [22-24].

BRAF has only MEK as a substrate for activation [6], and as discussed cutaneous cell lines with the *BRAF*^*V600E*^ mutation frequently have high sensitivity to MEK inhibitors *in vitro*[7]. However, patients with *BRAF*^*V600E*^ mutant cutaneous metastatic melanoma enrolled in clinical trials testing MEK inhibitors [25,26] have lower response rates than the use of the type I BRAF inhibitors vemurafenib or dabrafenib (GSK2118436) in the same population [11,13,27]. The reason for this discrepancy between *in vitro* and *in vivo* results with MEK inhibitors is not clearly understood at this time, but it may be related to a lower therapeutic window of MEK inhibitors in the clinic compared to type I BRAF inhibitors. This could be explained by the paradoxical activation of the MAPK pathway in *BRAF* wild type cutaneous cells, where type I BRAF inhibitors increase (or do not change) MAPK signaling in normal cells, while they efficiently block the MAPK pathway downstream of oncogenic BRAF^V600^. On the contrary, MEK inhibitors can equally block the MAPK pathway downstream of both oncogenic and wild type BRAF. This lack of differentiation most likely causes the dose limiting toxicities (DLT) at exposures *in vivo* that do not adequately block the MAPK pathway in *BRAF*^*V600*^ mutant melanoma. Despite this, MEK inhibitors are likely to have a role in the treatment of cancers with constitutive MAPK signaling from oncogenic mutations upstream of MEK. In particular the combination of MEK and RAF inhibitors may be beneficial by inducing higher MAPK inhibition in mutant cells and therefore lowering the cancer escape mechanisms and also decreasing toxicities from paradoxical MAPK activation [28], such as the development of cutaneous squamous cell carcinomas [29].

The majority of uveal melanomas bear a mutually exclusive activating mutation in either *GNAQ* or *GNA11*, resulting in overlapping functions in melanoma cells with the constitutive upregulation of the MAPK pathway [5]. In preclinical models it was shown that at least the *GNAQ* mutation resulted in sensitivity to downstream blocking of the MAPK pathway with a MEK inhibitor [4]. Our data demonstrating the sensitivity of uveal melanoma cell lines to TAK733 provides further evidence that it may be a clinical strategy to use MEK inhibitors to treat metastatic uveal melanomas. However, the same issues of a lack of correlation between the *in vitro* and clinical results when blocking oncogenic MAPK signaling using MEK inhibitors may apply to uveal melanomas.

The differential uptake of ^3^H-radiolabeled compounds that are trapped intracellularly upon metabolic processing allows testing their potential future use as PET probes in the clinical development of a new agent. It is anticipated that these radiolabeled metabolic probes can provide non-invasive pharmacodynamic information with the use of clinical PET scanners. In our studies, the highly sensitive cell lines had a decrease in the uptake of radiolabeled thymidine and deoxy-glucose that seemingly correlated with the cell viability and cell cycle results. However, there were variable changes in the highly resistant cell lines that did not directly correlate with the cell viability assay results (ex. M263 with marked decrease in the uptake of both tracers despite its resistance to TAK733). The metabolic tracer uptake studies were performed at a slightly earlier time point than the proliferation/viability assays to capture earlier events, and may be the reason of the discrepancy in results. These results raise the point that earlier PET scans with these tracers to detect early pharmacodynamic changes may not fully predict the later restaging imaging CT scan results.

In conclusion, inhibition of oncogenic MAPK signaling through MEK1 and MEK2 by TAK733 results in antitumor activity *in vitro* against a large subset of melanoma cell lines. We confirmed the previously reported cytotoxic effect of a MEK inhibitor against cell lines with *BRAF*^*V600E*^ mutations, but in addition the cytotoxic activity was evident in a high proportion of melanoma cell lines with *NRAS, GNAQ* or *GNA11* driver mutations. The antiproliferative and cell metabolism effects of this MEK inhibitor against melanoma cell lines can be detected with metabolic probes that could be tested with caution in the clinical development of this agent using PET imaging.

## Material and methods

### Reagents and cell lines

TAK-733 was obtained under a materials transfer agreement (MTA) from Millennium Pharmaceuticals, Inc. (Cambridge, MA) and dissolved in dimethyl sulfoxide (DMSO, Fisher Scientific, Morristown, NJ) to a stock concentration of 10 mM. The cutaneous melanoma cell lines of the M series were established from biopsies of metastatic melanoma of cutaneous origin as previously described [9] under the UCLA IRB approval #02-08-067 following the Declaration of Helsinki. SKMEL28, Wn1366 and SBCL2 were obtained from the American Type Culture Collection (ATCC, Rockville, MD). The uveal melanoma cell lines of the Mel20 series were established from fine needle aspirates of primary uveal melanoma lesions or from a metastatic uveal melanoma lesion (Mel20-09-196), obtained under the UCLA IRB approval #04-12-084. In the case of uveal melanoma cell lines, cells were cultured in DMEM with L-glutamine and 4.5 g/liter glucose (Mediatech Inc., Manassas, VA) containing 10% (unless noted, all percentages represent volume to volume) fetal bovine serum (FBS, Invitrogen, Carlsbad, CA) and 1% penicillin, streptomycin and amphotericin (Omega Scientific), with the addition of 5 μg/ml of bovine insulin (Sigma-Aldrich, St. Louis, MO). All cell lines were mycoplasma free when periodically tested using a Mycoalert assay (Lonza, Rockland, ME).

### Oncogenic analysis of cell lines

Cell lines were analyzed for known oncogenic activating mutations and deletions using multiplex PCR as well as by MALDI-TOF mass spectrometry (Sequenom, San Diego, CA) [30]. Point mutations were confirmed by PCR and direct sequencing as previously described [9]. In addition, most cell lines were analyzed by SNP arrays with DNA extracted from the cell lines hybridized onto Illumina Beadchip Human Exon 510 S-Duo (Illumina Inc., San Diego, CA).

### Cell proliferation and viability assays

Melanoma cell lines were treated with TAK-733 or parallel DMSO vehicle control at the given concentrations for 72 hours. Cell viability was measured using a tetrazolium compound [3-(4,5-dimethylthiazol-2-yl)-5- (3-carboxymethoxyphenyl)-2-(4-sulfophenyl)-2 H-tetrazolium (MTS)-based colorimetric cell proliferation assay (Promega, Madison, WI) as previously described [9].

### Cell cycle analysis

Cells were treated with different concentrations of TAK-733 (50 and 500 nM) or parallel vehicle control for 48 hours, fixed by Cytofix/Cytoperm solution and washed by Perm/Wash buffer according to fixation and pereabilization method recommended by BD bioscience, and then stained in sterile PBS containing 1.0% albumin bovine serum, 0.1% Nonidet P-40 (Sigma-Aldrich) and 3 μM DAPI (4',6-diamidino-2-phenylindole). Flow cytometry was analyzed using FlowJo (Tree Star Inc, Asland, OR).

### Western blotting

Western blotting was performed as previously described [31]. Primary antibodies included pAkt (Ser473), pAkt (Thr308), Akt, pS6K (Thr389), S6K, pS6 (Ser235/236), S6, pMEK (Ser217/221), MEK, pERK1/2 (Thr204/205), and ERK (all from Cell Signaling Technology, Danvers, MA), and α-actin (Sigma-Aldrich). Immunoreactivity was revealed using the ECL kit (Amersham Biosciences Co, Piscataway, NJ).

### *In vitro* metabolic tracer uptake assay

3 x 10^4^ cells/well were plated on 0.001% poly-L-lysine (Sigma-Aldrich) pre-incubated filter bottom 96-well plates (multiscreen HTS GV 0.22 μm opaque, Millipore, Billerica, MA) and rested for 24 hours. 0.1 and 1 μM of TAK733 or parallel DMSO vehicle control were added in triplicate for 20 hours. Cells were incubated for 1 hour with 2.0 μCi with metabolic tracers chosen as analogues of PET tracers: ^3^H-DDG (American Radiolabeled Chemicals Inc., St. Louis, MO) in glucose-free RPMI 1640 (Invitrogen), or methyl-^3^H-thymidine (thymidine, Moravek Biochemicals Inc., Brea, CA) in RPMI 1640. Extracellular metabolic tracer was washed off using a multiscreen HTS vacuum manifold system (Millipore). 100 μL scintillation fluid (Perkin Elmer, Waltham, MA) was added to each well and tritium count was measured on a 1450 microbeta trilux microplate (Perkin Elmer).

## Competing interests

Erika von Euw, Mohammad Atefi, Narsis Attar, Connie Chu, Sybil Zachariah, Barry L. Burgess, Stephen Mok, Charles Ng, Deborah J.L. Wong, Bartosz Chmielowski, Richard C. Koya, Tara A. McCannel: Have no competing interests. David I. Lichter, Elena Izmailova: Are employees of Millennium Pharmaceuticals, Inc., the manufacturer of TAK733. Antoni Ribas: Is a compensated ad hoc advisor to Millennium Pharmaceuticals, Inc., the manufacturer of TAK733.

## Authors’ contributions

EE, MA, NA, CC, SZ, BLB, SM, CN, DJLW, DIL, RCK: Performed experiments. EE, MA, TAM, EI, AR: Designed the studies. BLB, BC, EI, AR: Provided key reagents. EE, MA, AR: Wrote the manuscript. EE, MA, NA, CC, SZ, BLB, SM, CN, DJLW, BC, DIL, RCK, TAM, EI, AR: All authors approved the final manuscript.

## Supplementary Material

Additional file 1 **Figure S1**TAK733 MTS-based colorimetric cell proliferation assay curves in melanoma cell lines of cutaneous origin according to their BRAF (A) or NRAS (B) mutational status, WT (C) and of uveal origin (D). Modulation of the melanoma cell line viability at a range of different concentrations of TAK733. The effects of TAK733 on cell growth and viability were analyzed after 72 hours of treatment using an MTS assay. Click here for file

Additional file 2 **Figure S2**Time-course analyses of the effects of TAK733 on the signaling of the MAPK and PI3K/AKT pathways by Western blot. Two *BRAF*^*V600E*^ melanoma cell lines were exposed for varying time points to TAK733. A) The sensitive *BRAF*^*V600E*^ mutated cutaneous melanoma cell line M229; B) The resistant *BRAF*^*V600E*^ mutated cutaneous melanoma cell line M233. Click here for file
