# Supplementary material for: Antitumor effects of the investigational selective MEK inhibitor TAK733 against cutaneous and uveal melanoma cell lines
Source: Mol Cancer. 2012 Apr 19;11:22. doi: 10.1186/1476-4598-11-22 (PMC3444881; doi:10.1186/1476-4598-11-22)
Supplement: Additional file 1 — Figure S1TAK733 MTS-based colorimetric cell proliferation assay curves in melanoma cell lines of cutaneous origin according to their BRAF (A) or NRAS (B) mutational status, WT (C) and of uveal origin (D). Modulation of the melanoma cell line viability at a range of different concentrations of TAK733. The effects of TAK733 on cell growth and viability were analyzed after 72 hours of treatment using an MTS assay. [file 1476-4598-11-22-S1.ppt]

## Slide 1
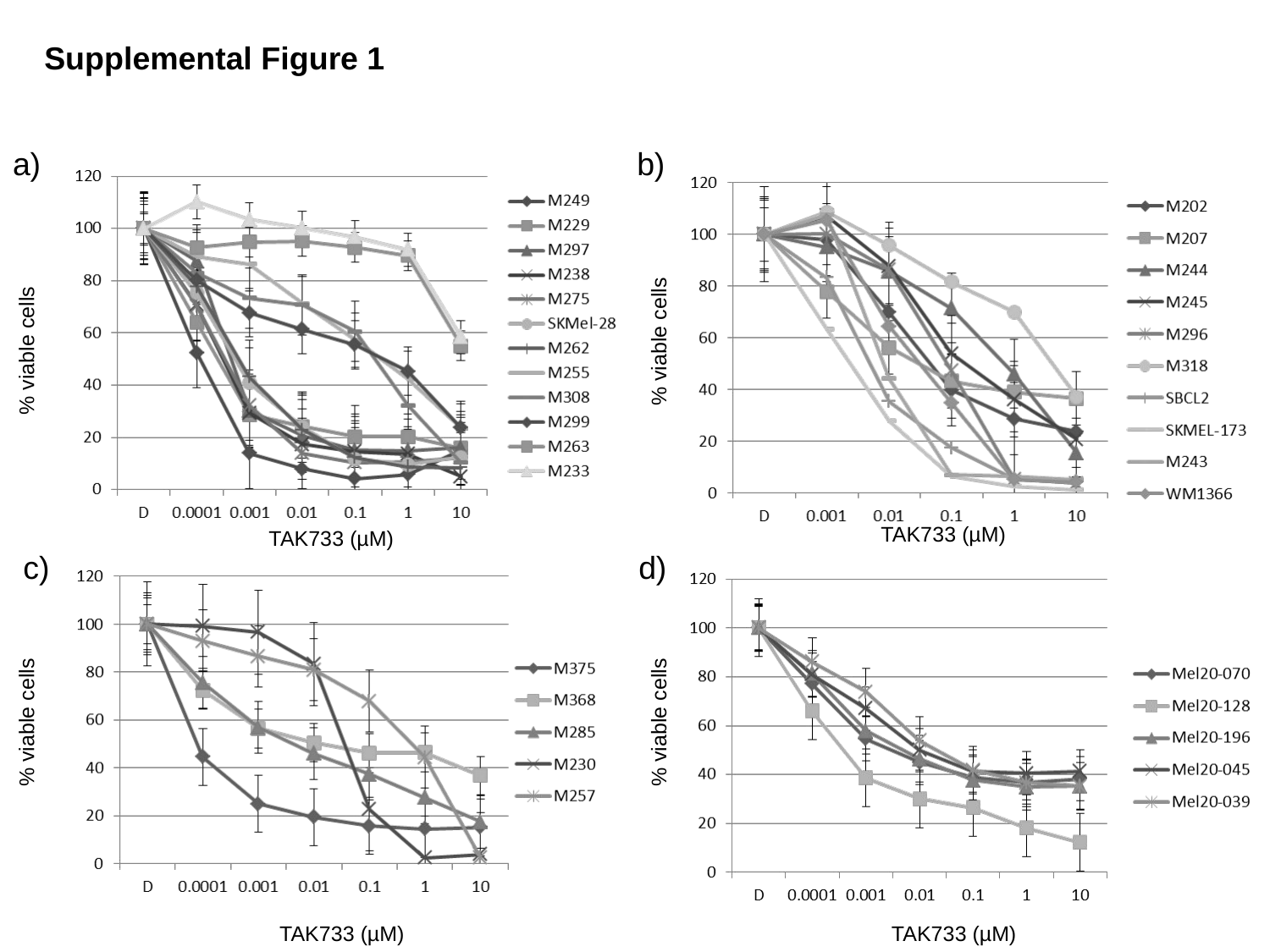

Supplemental Figure 1
a)
b)
% viable cells
% viable cells
TAK733 (µM)
TAK733 (µM)
c)
d)
% viable cells
% viable cells
TAK733 (µM)
TAK733 (µM)
