# Supplementary figures and images for: Antitumor effects of the investigational selective MEK inhibitor TAK733 against cutaneous and uveal melanoma cell lines
Source: Mol Cancer. 2012 Apr 19;11:22. doi: 10.1186/1476-4598-11-22 (PMC3444881; doi:10.1186/1476-4598-11-22)

## Slide 1
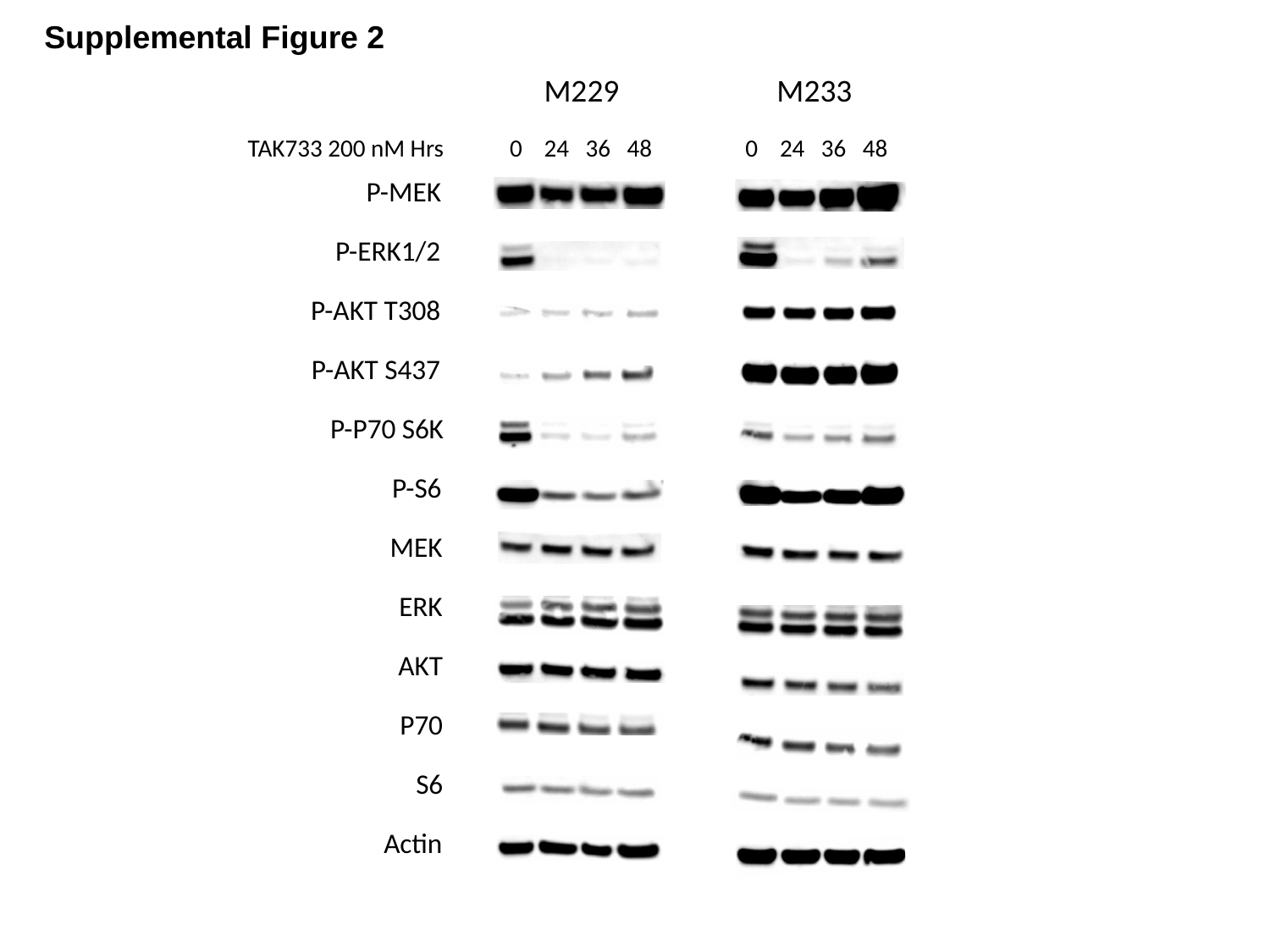

Supplemental Figure 2
M229
M233
TAK733 200 nM Hrs 0 24 36 48 0 24 36 48
P-MEK
P-ERK1/2
P-AKT T308
P-AKT S437
P-P70 S6K
P-S6
MEK
ERK
AKT
P70
S6
Actin

Supplement: Additional file 2 — Figure S2Time-course analyses of the effects of TAK733 on the signaling of the MAPK and PI3K/AKT pathways by Western blot. Two BRAFV600E melanoma cell lines were exposed for varying time points to TAK733. A) The sensitive BRAFV600E mutated cutaneous melanoma cell line M229; B) The resistant BRAFV600E mutated cutaneous melanoma cell line M233. [file 1476-4598-11-22-S2.ppt]
